# Anti-adrenergic effects of ranolazine in isolated rat aorta

**DOI:** 10.1186/cc13374

**Published:** 2014-03-17

**Authors:** P Marchio, MD Mauricio, FB El Amrani, S Guerra, D Aguirre-Rueda, SL Vallés, JM Vila, M Aldasoro

**Affiliations:** 1 University of Valencia, Spain

## Introduction

Ranolazine, a piperazine derivative, is used as an anti- anginal drug to treat patients with chronic angina in clinical practice [1] and may improve coronary blood flow by reducing compression effects of ischemic contracture, and by improving endothelial function [2],[3]. In the present study we investigate the vascular effects of ranolazine on the endothelium, adrenergic system and Ca^2^+ in isolated rat aorta.

## Methods

Rat aortic segments (3 mm long) with and without endothelium were mounted for isometric tension recording in organ baths containing Krebs-Henseleit solution. Electrical field stimulation (2, 4 and 8 Hz, 20 V, 0.25 ms duration for 30 seconds) was provided by a Grass S88 stimulator via two platinum electrodes positioned on each side and parallel to the axis of the aortic segment. Concentration- response curves of ranolazine (10^−7 ^to 10^−4 ^M) were obtained in a cumulative manner using endothelin-1, noradrenaline, thromboxane A2 and KCl as constrictor agents.

## Results

The contractile responses to electrical field stimulation were abolished by tetrodotoxin, guanethidine and prazosin, indicating that the contractile effect is due to the action of noradrenaline on alpha adrenoreceptors. Ranolazine diminished (*P *< 0.05) neurogenic adrenergic contractions induced by electrical field stimulation in aortic rings with and without endothelium. Ranolazine produced concentration-dependent relaxation in rings precontracted with noradrenaline (Emax 86 ± 6%, *n *= 10; *P *< 0.05) but not in rings precontracted with endothelin-1, thromboxane A2 and KCl. Neither L-NAME (10^−4 ^M), an inhibitor of nitric oxide synthase, nor indomethacin (10^−5 ^M), an inhibitor of cyclooxygenase, modified the relaxation induced by ranolazine. The calcium antagonist nifedipine (10^−6 ^M) reduced the relaxation induced by ranolazine.

## Conclusion

These results indicate that ranolazine diminished the contractile response induced by adrenergic stimulation, suggesting an effect as an adrenergic blocker. The relaxant effects of ranolazine on rat aortic vessels is not dependent on the endothelium-derived factors (nitric oxide or dilator prostanoids) but involves an interference with the entry of calcium through dihydropyridine calcium channels.
